# Editorial

**Published:** 1841-09-25

**Authors:** 


### THE MEDICAL EXAMINER.

PHILADELPHIA, SEPT. 25, 1841.

### BOSTON JOURNAL OF AUGUST 18.

Our attention has been called within a few days past to a critique on the case of President Harrison, and to an editorial article accompanying it, contained in the Boston Journal for the 18th of August. As to the anonymous critique, we entertain but one opinion of such attacks; it is morally and professionally wrong to censure the treatment pursued by a professional brother, except where an obvious good will result, and then the criticism should be conducted in a scientific way, and not to gratify envious or malicious feelings. Comments upon the case of President Harrison can be of little service, even if they were written in a tone indicative of a correct and honourable feeling, because the disease was evidently one of those obscure and complicated varieties in which the rules of treatment cannot be laid down with absolute certainty, and the management must be left in a great degree to the tact and skill of the attendant. The rules for treatment in such cases are more negative than positive; and of these negative rules there is none more certain than that depletion must be much more moderate than in ordinary frank, or inflammatory pneumonia. In the second place, the position of President Harrison was such that it would be hardly possible to discuss points of treatment, without exciting feelings which are least conducive to correct conclusions.

Under these circumstances we entirely approve of the course of Dr. Miller in abstaining from replying to these anonymous comments.

We must confess that we regret, extremely, the publication of the editorial comments. Knowing, as we do, that the general course of the editor of the Boston Journal is not fitted to wound the feelings of his brethren, or to countenance in any way an unfounded aspersion, we need not express our surprise at the following most uncalled for remarks.

*Report of the Treatment of the late President Harrison.*—A communication appears in the Journal to-day, that will be regarded with attention, it is apprehended, by practitioners generally. The circumstance of receiving the manuscript brings to mind an inquiry made some time since by an intelligent gentleman, whether the attending physician's account of the manner of treating the disease of which General Harrison died, was drawn up by himself? Now it cannot very well be concealed, since rumour has taken control of the story, that very many conceive that the scientific supervision of a medical gentleman in Philadelphia was thought quite necessary to give completeness to a report, which had been roughed out at Washington, but was thus finished according to modern requirements of literature and science. Some people, it is well known, are not satisfied with relating a discreditable fact, without giving it some important additions which might not be improbable; hence the suggestion that the prescriptions in the medical report were constructed cautiously, under the vigilant supervision of a scholar, some time after the death of the illustrious patient. If we could ascrtain the truth of the matter, it would be exceedingly gratifying. Not knowing, with certainty, whether envy or ignorance is at the bottom of these reports, a hope is entertained that those who can, will clear up the mist that now envelopes a matter in which the whole profession feels an interest.

As to the first charge or insinuation, we would state that the report was sent by Dr. Miller directly to us, and was not even altered to the degree which is perfectly justifiable without interfering with the tenure of the article. It was not touched, except some insignificant verbal changes, which every proofreader feels himself bound to make. The report carries with it internal evidence of not being got up; it was evidently not originally intended for publication, but was merely printed after its publication had been asked for. As to the implied statement that the prescriptions were altered by the author, his character and that of the consulting physicians is more than sufficient to shield them from insinuations of so contemptible a nature.

We have felt ourselves bound to notice this subject, partly because the report in question originally appeared in the columns of the Examiner, and partly from warm personal regard for men whose whole professional career has been pure and honourable, and who are now assailed with unfair censure and, still worse, with indirect insinuations of the most offensive character. But we have another, and we trust a higher reason; it is one which is deeply connected with the ethics of medicine. The publication of a case which terminated fatally and excited so much sympathy, requires no little moral courage on the part of the physician, and we conceive that it merits the respect of his professional brethren, and not ill-timed and ungenerous criticisms, or, what is far worse, doubts as to his professional honour.

